# Health problems in agricultural workers occupationally exposed to pesticides

**DOI:** 10.47626/1679-4435-2020-532

**Published:** 2021-02-11

**Authors:** Mariana Portela de-Assis, Raquel Cristine Barcella, Janaína Chiogna Padilha, Hildegard Hedwig Pohl, Suzane Beatriz Frantz Krug

**Affiliations:** Programa de Pós-Graduação em Promoção da Saúde, Universidade de Santa Cruz do Sul, Santa Cruz do Sul, RS, Brazil

**Keywords:** occupational health, occupational exposure, agricultural workers, pesticides

## Abstract

Agricultural workers are susceptible to several health problems as a result of occupational exposure to toxic substances, especially pesticides. The aim of this review was to describe the health issues associated with occupational exposure to pesticides in agricultural workers. A descriptive study, in the form of an integrative literature review, was conducted based on articles retrieved from the LILACS, SciELO and PubMed databases, published between January 2015 and October 2018. The searches were conducted using the keywords “pesticides,” “workers’ health,” “occupational exposure” and “agricultural workers.” The study was guided by the following research question: what health problems do agricultural workers experience as a result of occupational exposure to pesticides? The screening process led to the selection of 35 studies performed in several countries and continents, all of which shed light on the vulnerability of agricultural workers, especially due to the misuse of personal protective equipment and lack of knowledge about the correct use of these devices. The studies investigated a variety of health issues, and most reported a positive association between these conditions, which include cancer, and the use of pesticides. Educational and preventive measures must be implemented to promote the health of rural workers. Furthermore, it is crucial that governments play an active role in these initiatives and provide alternatives to pesticides for pest control.

## INTRODUCTION

Most health-related initiatives in Brazil are linked to the Unified Health System (SUS), which oversees and regulates these practices through ordinances and public policies. Issues associated with occupational health are guided by the National Workers’ Healthy Policy, established by Ordinance No. 1.823, issued August 23, 2012.^1^ As can be gleaned from its publication date, this policy has not been in place for long. Occupational health surveillance is an important tool for addressing workers’ health issues, since it provides an understanding of the occupational environment and the risk factors to which workers are exposed in their professional activities, all of which can be harmful to their health. These observations underscore the need for interventions to improve workplace health and safety. As such, workers’ health surveillance contributes to the promotion of interdisciplinary strategies to improve quality of life at work.^2^

Agriculture plays an important role in the economy of most countries and in the lives of local populations, since it provides stable jobs and a steady income to many families.^3^ Yet agricultural workers are also exposed to several health hazards as a result of their professional activities, including ultraviolet radiation; exhaust toxicity; inhalation of organic dust from spores and minerals when handling feed; exposure to microorganisms such as viruses, bacteria and infectious parasites, and their toxic products; as well as pesticides, which are among the greatest potential hazards to the health of agricultural workers.^3,4^

Pesticides comprise over one thousand chemical compounds used in agriculture to prevent, eliminate or control insects, weeds and fungal diseases.^5,6^ Given the widespread use of these substances to increase productivity, reduce the need for labor and manage plant diseases, all agricultural workers are likely to have at least some exposure to pesticides during their occupational activities. Pesticide contamination can have acute and chronic effects on exposed individuals, ranging from mild toxicity to neurotoxicity and even death.^7^ Pesticides have been associated with neurological, endocrine, psychological, immunological, respiratory, hematological, skin, kidney and liver issues, as well as fetal malformation.^8^ In tropical countries, pesticides can remain in the soil and water for a period of 1 to 2 months depending on temperature, sunlight and the presence of microorganisms, increasing the risk of contamination for workers and all other individuals who live in areas where these products are used.^4,9-11^

Brazil is one of the largest consumers of pesticides in the world, accounting for 86% of the pesticides used in Latin America.^8,12,13^ Currently, the country is experiencing significant changes in this sector due to the approval of Bill No. 6.299/2002 by the Chamber of Deputies. The bill modifies the criteria for approval, risk analysis and nomenclature of pesticides, allowing the indiscriminate use of pesticides, to the detriment of population health and in sharp contrast with the experience of developed countries in the European Union as well as the United States.^12,14^ The State has the duty to provide the population with comprehensive, universal and equitable health care. As such, it is also responsible for the oversight of initiatives implemented by employers in order to protect workplaces and residences. The environment can have a significant effect on workers’ physical, mental and social well-being. A major issue that affects the environment, in addition to health and work, is pesticide use and its repercussions on worker health.^15^

Given the importance of this issue and the need to understand the health impact of pesticides, we performed a search of the national and international literature to identify studies that discuss the health effects of occupational exposure to pesticides in agricultural workers.

## METHOD

An integrative literature review was conducted based on articles in journals indexed in the LILACS, SciELO and PubMed databases, published between January 1, 2015 and October 31, 2018. The searches were carried out using the following keywords: pesticides, workers’ health, occupational exposure and agricultural workers. This study was guided by the following research question: what health problems do agricultural workers experience as a result of occupational exposure to pesticides? Data were collected in October 2018. The inclusion criteria were scientific articles with full-text available online and open access publication in Spanish, English and Portuguese. Duplicate articles and those that did not address the research question were excluded from the review. The study selection process involved screening the titles of the studies retrieved by the literature search, followed by abstract screening and, if necessary, full-text reading to exclude any studies that did not cover the topic of the present review. The articles selected were then read in full and analyzed descriptively.

## RESULTS AND DISCUSSION

**Figure 1:**
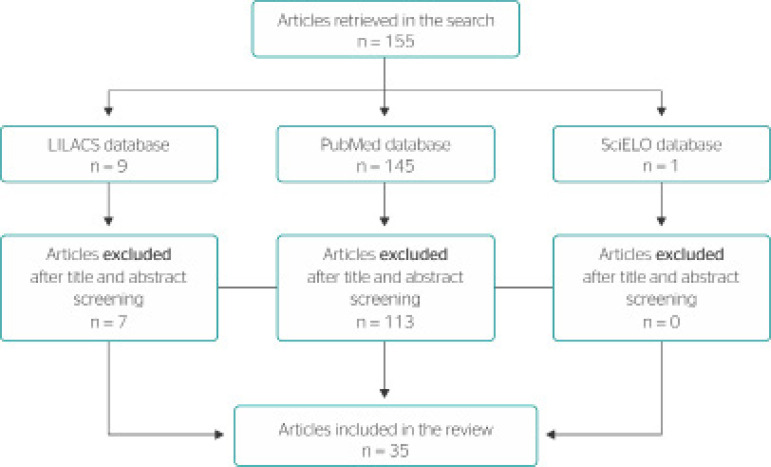
Study selection flow diagram and articles included in the review.

The database search retrieved 155 articles with the selected keywords. The study selection flow diagram is shown in Figure 1.

The data were extracted and entered into Table 1, which contains information on the following characteristics of the articles reviewed: authors, title, objectives, methodological design and main findings.

**Table 1:** Articles included in the review.

Author/year	Title	Objectives	Methodological design	Main findings
Aroonvilairat et al.^16^	Effect of pesticide exposure on immunological, hematological and biochemical parameters in Thai orchid farmers - a cross-sectional study	To assess the immunological, hematological and biochemical parameters of Thai orchid farmers who had frequent exposure to pesticides.	Cross-sectional study	Some biochemical parameters were altered in orchid farmers, pointing to the need for greater caution in the handling of pesticides.
Avgerinou et al.^17^	Occupational, dietary, and other risk factors for myelodysplastic syndromes in western Greece	To investigate possible risk factors for the development of myelodysplastic syndromes (MDS) in western Greece.	Case-control study	Univariate analysis showed that the risk of MDS was associated with a family history of hematological malignancies or solid tumors; exposure to pesticides, insecticides and herbicides; increased weekly intake of meat and eggs; and increased alcohol consumption. Fruit intake, on the other hand, had a protective effect.
Baumert et al.^18^	Sleep apnea and pesticide exposure in a study of US farmers	To examine the association between pesticide exposure and sleep apnea in pesticide applicators from a rural population in the United States.	Case-control study	There appears to be a positive association between carbofuran exposure and sleep apnea, since the former preceded the latter in all cases examined.
Boulanger et al.^19^	Agricultural exposure and risk of bladder cancer in the agriculture and cancer cohort	To assess the role of a wide range of agricultural activities and tasks in determining the risk of bladder cancer	Prospective cohort study	A possible association was found between specific agricultural activities (such as pesticide use) and bladder cancer.
Campos et al.^20^	Exposure to pesticides and mental disorders in a rural population of southern Brazil	To determine the prevalence of common mental disorders and self-reported depression, and analyze their association with pesticide exposure in a rural population in southern Brazil.	Three-stage cross-sectional study using a standardized questionnaire	The sample consisted of 869 participants, of whom 840 filled out the questionnaire completely. The prevalence of common mental disorders was 23% and that of self-reported depression was 21%.
Da Silva et al.^6^	Pesticide use and self- reported health Symptoms Among Rice Farmers in Zanzibar.	To examine pesticide use by small-scale rice farmers in Zanzibar, and evaluate self-reported symptoms of pesticide exposure.		Farmers who did not use PPE were more likely to experience toxicity. There is a need for more intensive training in safe pesticide handling.
Kab et al.^9^	Parkinsonism signs and symptoms in agricultural pesticide handlers in Washington state	To examine the association of pesticide exposure to signs and symptoms of Parkinsonism.	Clinical and laboratory assessment of pesticide applicator before and after exposure.	Signs and symptoms were moderate or absent. Further studies on the issue are required. When present, the most common symptoms were excessive salivation and action tremor.
Kachuri et al.^21^	Cancer risks in a population-based study of 70,570 agricultural workers: results from the Canadian census health and Environment cohort (CanCHEC)	To examine cancer risk in agricultural workers using a national Canadian database	Prospective cohort study	Agricultural workers are exposed to carcinogens. They have an increased risk of hematopoietic, prostate and lip cancer, as well as melanoma and brain tumors.
Khan et al.^22^	Occupational exposure to pesticides and resultant health problems among cotton farmers of Punjab, Pakistan	To examine occupational exposure to pesticides due to the handling practices used by agricultural workers; assess the health effects of pesticide use; and identify determinants of health in agricultural workers in two cotton farming districts in Pakistan.	Cross-sectional study	A significant fraction (34%) of agricultural workers reported several symptoms of pesticide toxicity; the most common were irritation of the skin and eyes, headache and dizziness.
Koh et al.^23^	Exposure to pesticide as a risk factor for depression: a population-based longitudinal study in Korea	To investigate the association between depression risk and high vs low level exposure to pesticides in the rural population.	Longitudinal study	Depression was positively associated with exposure to pesticides over a 20-year period, with an OR of 2.35 and 95%CI.
Lemarchand et al.^4^	Cancer incidence in the AGRICAN cohort study (2005-2011)	To compare cancer incidence rates between the AGRICAN cohort and the general population.	Prospective cohort study	No significant differences were observed in overall cancer incidence, but the AGRICAN cohort showed an increased incidence of prostate, lip, brain, breast (in men) and ovarian (in women) cancer, as well as non-Hodgkin lymphoma and multiple myeloma.
Lewis-Mikhael et al.^24^	Organochlorine pesticides and prostate cancer, is there an association? A meta-analysis of epidemiological evidence	To investigate the association between exposure to specific organochlorine pesticides and the risk of prostate cancer.	Meta-analysis	The results of the pooled data analysis did not support an association between any specific organochlorine pesticides and the risk of prostate cancer.
Lyu et al.^25^	Case control study of impulsivity, aggression, pesticide exposure and suicide attempts using pesticides among farmers	To investigate the association between exposure to organophosphate pesticides and aggressiveness, impulsivity and suicide attempts.	Case-control study	Individuals with previous suicide attempts had higher impulsivity and aggressiveness scores, and a higher number of symptoms of organophosphate exposure than control participants.
Meyer et al.^26^	Pesticide exposure and risk of rheumatoid arthritis among licensed male pesticide applicators in the agricultural health study	To test for a positive association between rheumatoid arthritis and the pesticide use in an agricultural health study.	Prospective cohort study	The results provided evidence of an association between exposure to some pesticides and rheumatoid arthritis in pesticide applicators.
Miranda-Contreras et al.^27^	Efectos de la exposición ocupacional a plaguicidas sobre la calidad del semen en trabajadores de una comunidad agrícola del estado Mérida, Venezuela	To analyze whether occupational exposure to pesticides is associated with semen quality and whether the effects of chronic exposure to pesticides on semen quality vary according to age.	Case-control study	The results of the study proved that occupational exposure to pesticides is associated with alterations in sperm quality, and may compromise reproductive function in agricultural workers.
Moisan et al.^28^	Association of Parkinson's disease and its subtypes with agricultural pesticide exposures in men: a case-control study in France	To investigate the association between pesticide use and Parkinson's disease in agricultural workers.	Case-control study	Pesticide use was significantly associated with symptoms of Parkinson's disease in men.
Muñoz-Quezada et al.^29^	Exposure to organophosphate (OP) pesticides and health conditions in agricultural and non-agricultural workers from Maule, Chile	To investigate pesticide exposure and health status in agricultural workers in the region of Maule, Chile.	A questionnaire was used to assess individuals with and without pesticide exposure.	Government regulations regarding the activity and training of agricultural workers must be implemented. A total of 56% of participants exposed to pesticides presented symptoms of poisoning at some point in time.
Muñoz-Quezada et al.^30^	Chronic exposure to organophosphate (OP) pesticides and neuropsychological functioning in farm workers: a review	To identify, assess and summarize available evidence on the neuropsychological effects of chronic exposure to organophosphates in rural workers.	Systematic review	Thirty-three articles met eligibility criteria for the review. Twenty-four of these found an association between chronic occupational exposure to organophosphates and poor neuropsychological performance in workers.
Negatu et al.^31^	Occupational pesticide exposure and respiratory health: a large-scale cross-sectional study in three commercial farming systems in Ethiopia	To verify whether pesticide exposure affects the respiratory health of agricultural workers and farmers in commercial farming systems.	Two-stage cross-sectional study	Individuals exposed to pesticides had a higher risk of health issues such as chronic cough and shortage of air, despite the short duration of exposure.
Ngowi et al.^32^	Pesticide health and safety challenges facing informal sector workers: a case of small-scale agricultural workers in Tanzania	To describe the health and safety challenges associated with pesticide use faced by small-scale agricultural workers in Tanzania.	Literature review	In addition to occupational exposure to pesticides, agricultural workers may have to contend with contaminated food and water, which can also lead to toxicity. Additional findings included reckless use of pesticides; unfamiliarity with the negative effects of these products; rampant underreporting of acute pesticide toxicity and the need to implement public policies to improve the health of small-scale agricultural workers.
Piccoli et al.^13^	Pesticide exposure and thyroid function in an agricultural population in Brazil.	To investigate the association between thyroid hormone levels and agricultural practices, current use of pesticides and serum pesticide levels in a rural population.	Randomly sampled cross-sectional study	Findings showed that both cumulative and recent exposure to agricultural pesticides can affect thyroid function, producing similar symptoms to those associated with hypothyroidism, especially in men.
Piel et al.^5^	Central nervous system tumors and agricultural exposures in the prospective cohort AGRICAN	To investigate the association between the incidence of central nervous system tumors and agricultural exposures, pesticide use and livestock rearing.	Prospective cohort study	Increased risk of different types of cancer depending on the agricultural activity performed. The risk was also increased din workers who used pesticides.
Quandt et al.^33^	Olfactory function in Latino farmworkers over 2 years: longitudinal exploration of subclinical neurological effects of pesticide exposure	To compare olfactory function between Latino farmworkers and Latino workers in industries without pesticide exposure, assessing changes in olfaction over a 2-year period.	Case-control study	Pesticide exposure was associated with neurodegenerative markers such as olfactory loss.
Quansah et al.^34^	Associations between pesticide use and respiratory symptoms: a cross-sectional study in southern Ghana	To analyze the association between pesticide use and respiratory symptoms in Ghana.	Cross-sectional study	Significant positive associations were observed between fumigants and wheezing; fungicides and wheezing as well as phlegm production; insecticides and chronic cough as well as wheezing; and exposure to pesticides and respiratory symptoms.
Salerno et al.^35^	An Italian population- based case-control study on the association between farming and cancer: are pesticides a plausible risk factor?	To investigate the association between agriculture (used as a proxy for pesticide exposure) and cancer in suburban Vercelli (Italy).	Case-control study	The results suggest a possible association between pesticide exposure and cancer incidence.
Sankoh et al.^36^	An assessment of the impacts of pesticide use on the environment and health of rice farmers in Sierra Leone	To assess the prevalence of pesticide use among rice farmers in Sierra Leone, focusing on the different application methods and the evaluation of their impact and risks to human health and the environment.	A qualitative and quantitative study using a semi-structured questionnaire and interview	Skin issues, nausea, seizures, respiratory disorders, blurred vision, loss of appetite, lacrimation and nervous system disorders were significantly more frequent among farmers who used pesticides than those who did not.
Sekhotha et al.^37^	Exposure to agrochemicals and cardiovascular disease: a review	To assess the relationship between agrochemical formulations and cardiovascular illness in rural workers.	Non-systematic review	There is a close relationship between agrochemical formulations and cardiovascular illness.
Varona et al.^11^	Determining social factors related to pesticide poisoning among rice farmers in Colombia	To describe the circumstances of pesticide exposure and subsequent poisoning in agricultural workers.	Multimethod, multilevel study including ethnographic methods, surveys and measurement of pesticide levels in water and biological samples	Precarious working conditions increase the likelihood of pesticide exposure and lead to the exclusion of farmworkers from the occupational health system. Findings: pesticides detected in water samples, and workers presenting with mild (12.86%), moderate (67.98%) or severe toxicity (5.51%).
Zhang et al.^38^	Health effect of agricultural pesticide use in China: implications for the development of GM crops	To investigate the association of pesticides used on GM crops to blood chemistry parameters and peripheral nerve conduction in Chinese farmers.	Prospective cohort study	GM crops are likely to benefit a large number of agricultural workers in China and around the world by allowing for changes in pesticide use and the adoption of compounds that are less harmful to occupational health.
Zhang et al.^39^	Pesticide poisoning and neurobehavioral function among farm workers in Jiangsu, people's republic of China	To analyze the association between self-reported pesticide poisoning and neurobehavioral impairment in a group of agricultural workers in China.	Case-control study	The findings provided preliminary epidemiological evidence of the association between occupational exposure to pesticides and neurobehavioral function in Chinese agricultural workers.

PPE: personal protective equipment; GM: genetically modified; 95CI%: 95% confidence interval; OR: odds ratio.

Agricultural workers are exposed to a series of physical, chemical and biological health hazards during their professional activities. Pesticides are widely used to increase crop yield in these settings, but the absence of prior assessment, an effective cost-benefit analysis, and evaluations of the amount and time of application result in a significant risk to the health of workers. The lack of assessment of agricultural workers, combined with the negligent attitude of some governments, aggravate the risks of chemical exposure for agricultural workers, who are often unaware of these issues, much like the health care workers they eventually seek, often when their healthy is already severely compromised.^6,11,40,41^ Studies show that agricultural workers tend to consume less alcohol and tobacco than workers in other occupations; their work activities also include regular moderate-intensity physical activity, which contributes to lower morbidity and mortality rates than observed in the general population. Yet pesticide handling exposes these workers to a number of other illnesses, as demonstrated by studies performed in several countries where pesticides are used in agriculture.^4,21,30^

All of the articles included in this review evaluated the chemical risks to which agricultural workers are exposed, as they examined the consequences of exposure to pesticides during occupational activities. A study performed in Chile^30^ found that the pesticides most commonly used in vineyards were associated with health problems in farmers. Several disorders were identified in the sample, with acute poisoning being the most significant and prevalent. The most commonly reported symptoms were headaches, nausea, vomiting, shortness of breath, bradycardia, dermatitis, burns and eye irritation. Gesesew et al.^40^ studied agricultural workers in Ethiopia, and found that acute toxicity was also the main health problem in that population. In the study, agricultural workers expressed awareness of inhalation and ingestion as routes of exposure to pesticides, but were unaware of the possibility of contamination through the skin, which poses a major challenge for preventive measures. Additionally, agricultural workers who experienced symptoms of pesticide toxicity tended to resort to home remedies such as drinking milk and applying creams to the affected area, seeking health care services only in severe cases.

The inadequate use of pesticides can also have neurobehavioral effects. A case-control study conducted in China revealed that chronic occupational exposure to pesticides can lead to symptoms of depression, lack of motivation and anxiety.^39^ A systematic review^30^ found that exposure to organophosphates may affect cognitive and motor functions, as well as concentration, agility, memory and coordination. A study performed in Pakistan revealed that the symptoms most frequently cited by cotton farmers after pesticide exposure were irritation of the skin and eyes, headache and dizziness, followed by shortness of breath, vomiting and fever.^22^

China is considered a major global supplier of pesticides, in addition to making extensive use of these products in agricultural production. As such, the country has conducted a number of studies to investigate the short- and long-term effects of these substances on the population. A cohort study in which the hematological and neurological parameters of 246 agricultural workers from three Chinese provinces were evaluated over a 30-year period demonstrated that a longer duration of pesticide exposure can trigger alterations in nervous system functioning. Laboratory analyses also provided evidence of muscle damage. Short-term effects in the form of altered liver and kidney function were also observed within three days of pesticide exposure. Another relevant finding was that only 14% of those interviewed used personal protective equipment (PPE). The study concluded that both short- and long-term exposure were associated with hematological, hepatic and peripheral nervous system alterations.^41^

Alterations in hematological parameters due to pesticide use were also reported in Thai orchid farmers, especially those who did not use PPE. Toxicological analyses conducted after pesticide application revealed alterations in immunological factors - B lymphocyte numbers and biochemical marker levels - which were not clinically significant, but suggested caution and emphasized the need for PPE in these settings.^16^ In Greece, a case-control study of agricultural workers being treated for myelodysplastic syndrome found that occupational exposure to pesticides increased the risk of developing this condition.^17^

A study of rice farmers in Sierra Leone revealed that health issues such as dermatitis, nausea, seizures and respiratory illnesses were significantly more frequent among farmers who used pesticides than those who did not. The authors noted that pesticides are cheap and easily accessible in Sierra Leone, as they are smuggled into the country. The indiscriminate use of these substances has a significant impact on health and the environment, leading to soil and groundwater contamination, and reducing biodiversity. The study also found that the procedures used in the storage, application, handling and preparation of these products were detrimental to human health. Lastly, the health care workers interviewed in the investigation emphasized the lack of national programs to address health and environmental issues, as well as the absence of education and prevention initiatives in the country.^36^

The main determinants of pesticide exposure include lack of knowledge about correct pesticide use, the inability to comprehend product labels, inadequate storage conditions and the underestimation of the health risks of these products.^11,40^ The studies also showed that many rural workers do not routinely use PPE, which could protect them from exposure to health and safety risks during their occupational activities. Barriers to the use of this important preventive measure include low education levels, insufficient understanding of the importance of PPE and a lack of training, in addition to the high costs of specialized equipment.^5,6,10^ A study performed in Ethiopia found that 42% of agricultural workers had never used PPE.^40^ Ghafari et al.^3^ reported that 68% of rural workers in Iran did not use any form of PPE during pesticide application, while Zhang et al.^38^, in China, reported that only 13.4% of farmers used PPE when handling pesticides. The attention to correct PPE use, which includes removing clothing immediately after use and washing the hands and face after handling pesticides, are as important as the equipment itself.^5,40^ Acute toxicity can also be caused by inadequate storage and the reuse of empty pesticide containers to store water and food, as reported by Muñoz-Quesada et al.^30^, Gesesew et al.^40^ and Silva et al.^6^.

A study performed in Tanzania found that an estimated 220 thousand people die every year from pesticide exposure in low- and middle-income countries. The authors attribute this to lack of knowledge and low education, in addition to the absence of legislation to protect the health of agricultural workers in these locations. These factors contribute to poor handling practices that increase exposure to dangerous products, leading to toxicity and long-term effects on workers’ health. The study highlights the importance of continuing education regarding the effects of these products and their adequate use.^32^

The handling of pesticides also exposes workers to an increased risk of depression and other mental and behavioral disorders, in addition to higher levels of aggressiveness/impulsivity and suicide attempts, as shown in studies performed in China^25^ and Korea.^23^ According to the researchers, the exposure to high levels of organophosphate pesticides over several years, with or without episodes of toxicity, inhibits acetylcholinesterase, leading to the accumulation of acetylcholine in cholinergic receptors and decreasing impulse control, contributing to aggressive behavior, depression and suicidal ideation.

The frequency of sleep apnea was also higher in workers exposed to pesticides in an agricultural community in the United States. According to Baumert et al.,^18^ the inhibition of acetylcholinesterase induces respiratory depression, increasing the risk of this condition. Sleep apnea, in turn, contributes to increased morbidity and mortality in individuals exposed to pesticides, interfering with workers’ sleep and quality of life. The results of the study showed that in male agricultural workers, exposure to carbofuran was positively associated with sleep apnea.

Olfactory and hearing loss were studied by Quandt et al.^33^ and França et al.^8^, respectively. In a study of Latino workers exposed to pesticides in the United States, Quandt et al.^33^ found that pesticides can significantly affect olfactory function even when inhaled at low doses. The study also found that olfactory impairment occurs earlier than other symptoms of neurodegenerative diseases such as Parkinson’s. Since the study had a short follow-up period, it was not possible to determine whether the duration of exposure was associated with progressive olfactory loss, and it is therefore possible that factors other than pesticide exposure also contribute to the occurrence of this symptom. A separate study of tobacco farmers in Brazil^8^ also found that organophosphates can affect auditory function by inhibiting acetylcholinesterase and interfering with the transmission of action potentials from efferent fibers, thereby affecting peripheral, central and vestibular components of the auditory system. The issue is aggravated by the exposure to multiple risk factors for hearing loss, such as pesticides and noise, both of which are present in agricultural settings.

The association between pesticide use and Parkinson’s disease was investigated by Nielsen et al.^10^ in a study conducted in Washington, D.C, in the United States. Clinical and laboratory examinations performed before and after the period of exposure revealed little to no association between pesticide use and the development of the disease. However, these findings may have been influenced by the small sample size of the study. Additionally, factors such as living far from the place of work and correct PPE use may have had protective effects and prevented continuous exposure to the contaminant. A study of French agricultural workers, however, did identify an association between the intensity of pesticide exposure and tremors associated with Parkinson’s disease. Herbicides were the most common type of pesticide used by participants, followed by insecticides and fungicides.^28^

Some experimental studies suggest that the neurotoxicity caused by exposure to pesticides can induce endocrine alterations, especially in agricultural workers, who, in most cases, come into direct contact with these products. The study was performed in a city in southern Brazil, and its results showed that pesticide toxicity may be associated with mental disorders, since these are more frequent in individuals who use pesticides containing dinitroanilines, pyrethroids, sulfonylurea and aliphatic alcohol. The data obtained in this study also pointed to an association between early pesticide exposure and mental illness.^20^

A study of agricultural workers in North Carolina, in the United States, focused on the assessment of rheumatoid arthritis. Though few studies have examined the association between these variables, the study in question found that occupational exposure to some types of pesticide was linked to an increase in the prevalence of rheumatoid arthritis, regardless of age, smoking history and educational level. The incidence of rheumatoid arthritis was associated with the frequent use of the organophosphate insecticide fonofos, the carbamate insecticide carbaryl and the sulfonylurea herbicide chlorimuron-ethyl.^26^ In Venezuela, a study of sperm quality in agricultural workers exposed to pesticides confirmed the risk of infertility in male farmworkers exposed to these products.^27^

A non-systematic review of the relationship between pesticide particles and cardiovascular disease in agricultural workers also found a close association between these variables. However, the authors noted that further studies are needed to investigate the health hazards associated with pesticide exposure, and that governments and agricultural producers must implement measures to reduce cardiovascular mortality in this population.^37^ A study of agricultural workers in vineyards in southern Brazil revealed that both currently used and banned pesticides affect the thyroid gland, leading to symptoms similar to hypothyroidism, especially in men, who tend to be exposed to higher concentrations of pesticides than women.^13^

A study conducted in Ethiopia also identified an increased risk of respiratory illness in agricultural workers. The authors described their finding as ‘alarming,’ since the workers demonstrated significant reductions in pulmonary function - as evidenced by chronic cough and shortness of breath - despite the relatively short duration of pesticide exposure (4 years on average), alerting to the need for urgent intervention.^31^ Another study evaluated the association between pesticide exposure and wheeze in male participants of the Agricultural Health Study (AHS), a prospective investigation of farm workers in North Carolina and Iowa, in the United States. The results showed that 19 pesticides were significantly associated with allergic wheeze; 21 with non-allergic wheeze; and 11 were associated with both, suggesting that commonly used pesticides in agricultural and residential settings can have adverse respiratory effects.^42^

With regards to cancer, some authors note that rural workers are less likely to develop cancer than the general population, probably due to a lower intake of alcohol and drugs and the level of physical activity in their professional practice. However, some types of cancer are common in this population, either due to exposure to ultraviolet rays, as in the case of lip cancer and myeloma, or pesticide exposure, which is associated with a higher frequency of prostate and hematological cancer, as well as non-Hodgkin’s lymphoma and even brain tumors.^4,21,19^ Though a study of pesticide exposure and bladder cancer did not support a conclusive association between these variables, it did reveal a significantly higher incidence of cancer in vegetable farmers, women and non-smokers.^19^ A study of agricultural workers in France examined the association between cancer and exposure to some types of pesticides, but could not determine a causal association between these variables due to the high frequency with which workers moved between crops and their handling of multiple types of product. The study also identified an interesting association between pesticide use and an increase in the incidence of central nervous system tumors, especially gliomas and meningiomas.^5^ A case-control study of pesticide exposure which compared agricultural and non-agricultural workers in Italy over a lengthy chronological period (1965-2009) revealed a higher likelihood of cancer in the former group than in the latter. The association between pesticide use and non-melanoma skin cancer, colorectal and breast cancer also reached statistical significance.^35^

In female agricultural workers, studies showed an increase in the incidence of ovarian and pancreatic cancer, acute myeloid leukemia and breast cancer.^3,21^ The prevalence of breast cancer among the wives of agricultural workers who used pesticides on their farms was also studied, but no differences were observed between women who handled the pesticides and those who did not, nothing a similar breast cancer risk in both participant groups. The highest risk of breast cancer was observed in premenopausal women and those who used organophosphate pesticides.^43^ In a meta-analysis performed by Spanish researchers, the analysis of pooled data did not support an association between organochlorine pesticides and the risk of prostate cancer, suggesting the need for further studies in this area.^24^ In China, a study of genetically modified crop production found it to be associated with decreased pesticide use, as well as blood chemistry markers and peripheral nerve conduction. The results demonstrated that this type of crop requires less pesticide and allows for the use of products that are less hazardous to health, which could be greatly beneficial to agricultural workers.^24^

## CONCLUSIONS

The present review identified several studies that demonstrated the hazardous effects of occupational pesticide exposure on the health of agricultural workers around the world. There also appears to be some concern about the need to conduct further research on this issue. The articles included in this review identified several ways in which excessive pesticide exposure during occupational activities can harm the health of agricultural workers. The conditions associated with pesticide exposure include hematological alterations, respiratory issues, endocrine dysfunction, neurotoxicity, infertility and, most concerningly, an increased risk of some types of cancer.

The use of pesticides and their impact on the health of agricultural workers is a global concern. Governments should acknowledge their role as protectors of public health and create laws and public policies whose aim is not only to increase land productivity, but also promote the protection of workers against possible occupational risks. Studies that investigate the use of less hazardous chemical alternatives are also required. The encouragement of non-pesticide alternatives for pest and weed control would also have a major impact on the health of both producers and consumers of agricultural products, bringing balance and health to the environment and to agricultural workers.

Awareness campaigns, subsidies for the acquisition of PPE and specialized training are crucial for improving occupational safety and working conditions. Adequately trained workers are more likely to use PPE, implement protective measures and use safe handling practices. The labels on pesticides and other agricultural products must also be made more understandable, and workers must be encouraged to pursue formal education and seek basic health services; these steps will allow agricultural workers to access the knowledge and resources necessary to take preventive and effective measures to protect their health and that of their families. Of equal importance is the need to continue conducting short- and long-term studies of pesticides and the harm and illnesses they cause when they come into contact with the human body.
